# DBENet: Dual-Branch Encoder Network for brain MRI lesion segmentation

**DOI:** 10.3389/fneur.2026.1766271

**Published:** 2026-07-23

**Authors:** Qiang Zhao, Zhaohui Zhang, Tinghua Cao

**Affiliations:** 1 Department of Radiology, People's Hospital of Chongqing Liang Jiang New Area, Chongqing, China; 2 School of Computer Science, Qufu Normal University, Qufu, China; 3 Department of Radiology, Bishan District Traditional Chinese Medicine Hospital, Chongqing, China

**Keywords:** brain lesion, deep learning, Dual-Branch Encoder Network, segmentation, Spatial and Frequency Fusion

## Abstract

**Introduction:**

Accurate brain lesion segmentation in Magnetic Resonance Imaging (MRI) remains challenging due to heterogeneous lesion appearance, variable scales, and ambiguous boundaries.

**Methods:**

We propose DBENet, a Dual-Branch Encoder Network for brain MRI lesion segmentation. The spatial and frequency branches first extract lesion-related features from complementary domains. The Spatial and Frequency Fusion (SFF) module then integrates these features into a hybrid lesion representation. In parallel, lesion-aware prompts are encoded by the Segment Anything Model (SAM) prompt encoder to generate prompt embeddings. Finally, the Multi-scale Attention Fusion (MAF) module takes the hybrid representation and prompt embeddings as inputs, and progressively integrates them across scales for contextual modeling and boundary refinement.

**Results:**

Extensive experiments on the ISLES 2022 and BraTS 2018 datasets demonstrate that DBENet outperforms state-of-the-art approaches, achieving Dice scores of 0.8621 and 0.8261, respectively, while providing superior lesion localization and boundary delineation. Ablation studies confirm the complementary benefits of SFF and MAF.

**Discussion:**

Furthermore, DBENet achieves a favorable balance between segmentation accuracy and computational cost, supporting practical implementation under moderate hardware constraints. Future work will extend DBENet to volumetric 3D segmentation. We will also investigate multimodal learning and foundation-model adaptation to improve robustness across diverse imaging protocols.

## 1 Introduction

Brain lesion segmentation in Magnetic Resonance Imaging (MRI) is essential for diagnosing ischemic stroke and brain tumors, planning treatment, and assessing prognosis (1–3). In these diseases, lesion extent and boundary characteristics directly affect clinical interpretation. Precise delineation enables quantitative assessment of disease burden and progression. Such measurements also support therapeutic decision-making and longitudinal outcome monitoring. However, manual annotation remains labor-intensive and time-consuming. Inter-observer variability further limits scalability and reproducibility. These limitations have driven the development of accurate and reliable automated segmentation methods. Despite recent advances in deep learning-based medical image analysis (4, 5), accurate brain lesion segmentation remains a challenging task. Brain lesions exhibit substantial variations in size, shape, location, and appearance across different pathological conditions. For example, ischemic stroke lesions often show blurred boundaries, which arise from gradual transitions between infarcted tissue and surrounding brain parenchyma. In contrast, brain tumors frequently contain heterogeneous subregions, complex textures, and irregular contours. Furthermore, MRI images frequently suffer from low contrast, imaging artifacts, and noise, which further obscure lesion boundaries and increase segmentation difficulty.

**Figure 1:**
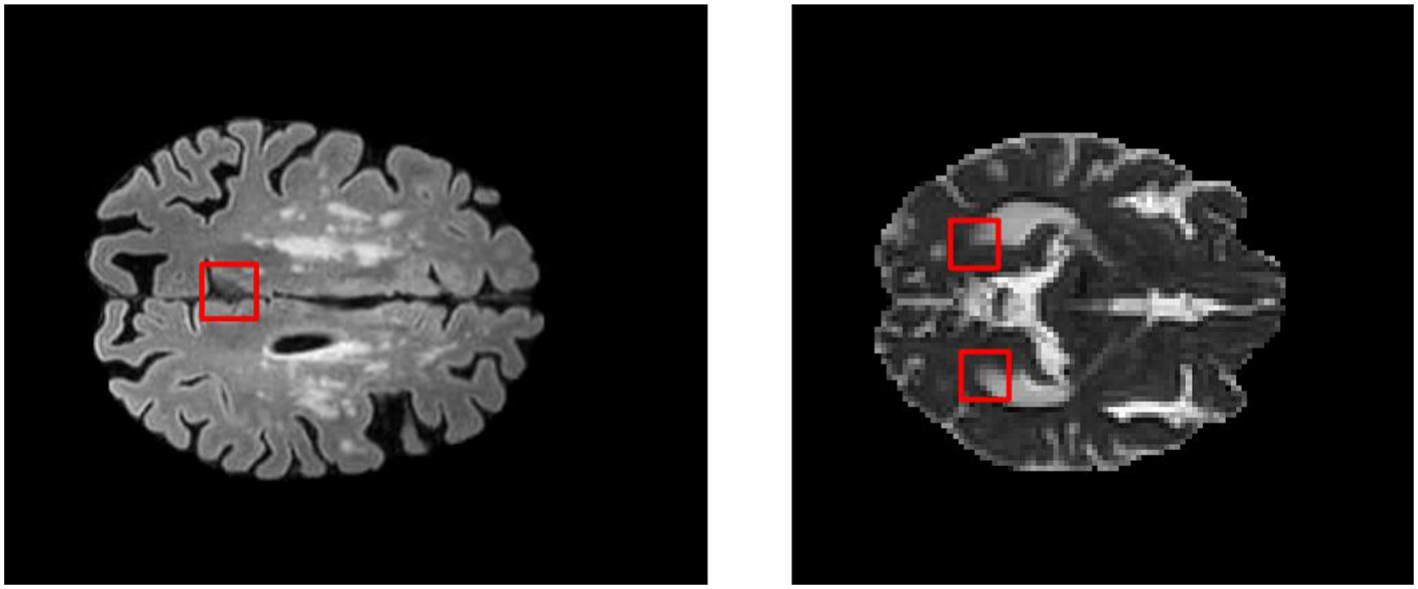
Examples of brain MRI images containing blurred lesion boundaries (highlighted by red rectangles). Such ambiguous regions are difficult to accurately delineate using conventional spatial-domain segmentation methods.

As illustrated in Figure 1, many brain lesions exhibit blurred or poorly defined boundaries, making them difficult to distinguish from surrounding tissues. Most existing segmentation networks primarily model spatial-domain intensity and texture patterns. However, these cues become unreliable when lesion boundaries are blurred, low-contrast, or corrupted by noise. Consequently, subtle structural details may be overlooked, leading to inaccurate boundary delineation and incomplete lesion segmentation.

Frequency-domain analysis provides a complementary perspective for characterizing lesion structures. Frequency transforms decompose MR images into low- and high-frequency components. Low-frequency components encode global anatomical structure, whereas high-frequency components preserve edges, textures, and boundary transitions. In particular, wavelet transforms possess excellent spatial-frequency localization capabilities and can effectively capture multi-scale structural characteristics (6–9). Through multi-resolution decomposition, wavelet-based representations preserve subtle edge information and texture patterns that may be weakened in the spatial domain, thereby providing richer contextual cues for identifying complex lesion boundaries.

Recent studies (10–12) have demonstrated that combining spatial and frequency representations can improve feature discrimination in medical image segmentation tasks. Spatial features are effective for modeling lesion morphology and contextual relationships, whereas frequency features can enhance the representation of fine-grained details and boundary information. Therefore, jointly exploiting these complementary characteristics offers a promising strategy for addressing the challenges posed by heterogeneous lesion appearances, low image contrast, and indistinct boundaries, ultimately leading to more accurate and robust brain lesion segmentation.

The main contributions of this work are summarized as follows:

- We propose DBENet, a dual-branch encoder network for brain MRI lesion segmentation. The framework combines spatial-frequency representation learning with multi-scale prompt-guided decoding, enabling more accurate delineation of lesions with blurred boundaries and heterogeneous appearances.
- We design a Spatial and Frequency Fusion (SFF) module to construct domain-complementary lesion representations. This module adaptively integrates spatial morphology with frequency-domain details, thereby enhancing boundary-sensitive features under low contrast and noisy imaging conditions.
- We introduce a Multi-scale Attention Fusion (MAF) module for prompt-guided segmentation decoding. The module aligns hybrid lesion features with Segment Anything Model (SAM) prompt embeddings across multiple scales, improving contextual consistency and boundary refinement.
- Extensive experimental results demonstrate that the proposed DBENet consistently outperforms representative segmentation methods across all evaluation metrics on the benchmark datasets.

## 2 Related work

Convolutional Neural Networks (CNNs) remain a dominant paradigm in medical image segmentation. Among them, U-Net and its variants, including Attention U-Net, Residual U-Net, ResUNet++, and Recurrent Residual U-Net, have demonstrated remarkable success in a wide range of medical image analysis tasks (13–17). These encoder-decoder architectures effectively combine high-level semantic information with low-level spatial details through skip connections, significantly improving segmentation accuracy. To further enhance feature representation, numerous studies have incorporated attention mechanisms into CNN-based architectures (18–21). Attention modules help segmentation networks prioritize lesion-relevant regions and suppress irrelevant background responses. However, most attention mechanisms still operate mainly in the spatial domain. They may overlook subtle frequency cues and blurred boundary details in low-contrast lesions.

Recently, Transformer-based architectures have attracted considerable attention in medical image segmentation due to their strong capability for capturing global contextual information (22–25). Unlike conventional convolutional operations that focus on local receptive fields, Transformers employ self-attention mechanisms to model long-range dependencies across the entire image. Motivated by their success in natural language processing and computer vision, researchers have introduced Transformer-based models into brain lesion segmentation tasks. For example, Lin et al. (26) proposed CKD-TransBTS, a clinical knowledge-driven brain tumor segmentation framework that exploits modality-correlated cross-attention to enhance multi-modal feature learning. Zhu et al. (27) developed a multimodal MRI segmentation framework that combines semantic segmentation and edge detection through Transformer-based feature extraction and fusion strategies. These studies demonstrate the strengths of Transformer architectures in modeling complex lesion characteristics. However, Transformer-based methods still mainly learn spatial-domain dependencies. They may inadequately preserve subtle frequency-domain cues, fine boundary details, and low-contrast lesion structures.

Brain lesion segmentation remains challenging across ischemic stroke and brain tumor imaging (28, 29). These lesions show substantial variations in size, shape, location, texture, and intensity distribution. Stroke lesions often exhibit diffuse boundaries and low contrast against surrounding tissues. Brain tumors frequently contain heterogeneous subregions with irregular structures and complex appearance patterns. These characteristics limit methods that mainly depend on spatial-domain representations. In particular, blurred boundaries and weak lesion contrast require complementary frequency-domain cues. Such cues can preserve fine structural details and boundary-related patterns that may be weakened in spatial feature learning.

To address these challenges, complementary representations beyond the spatial domain are needed. Frequency-domain features can preserve fine structural details and boundary-related patterns that may be weakened during spatial feature learning (11, 12). Meanwhile, multi-scale feature modeling is important for lesions with variable sizes, irregular shapes, and diffuse boundaries. Motivated by these observations, we propose DBENet to integrate spatial-frequency fusion with multi-scale attention refinement for brain lesion segmentation.

## 3 Method

In this section, we present the proposed DBENet in detail. We first describe the overall dual-branch architecture, followed by the Spatial and Frequency Fusion (SFF) and Multi-scale Attention Fusion (MAF) modules. Finally, we introduce the loss functions used for network optimization.

### 3.1 Overall architecture

**Figure 2:**
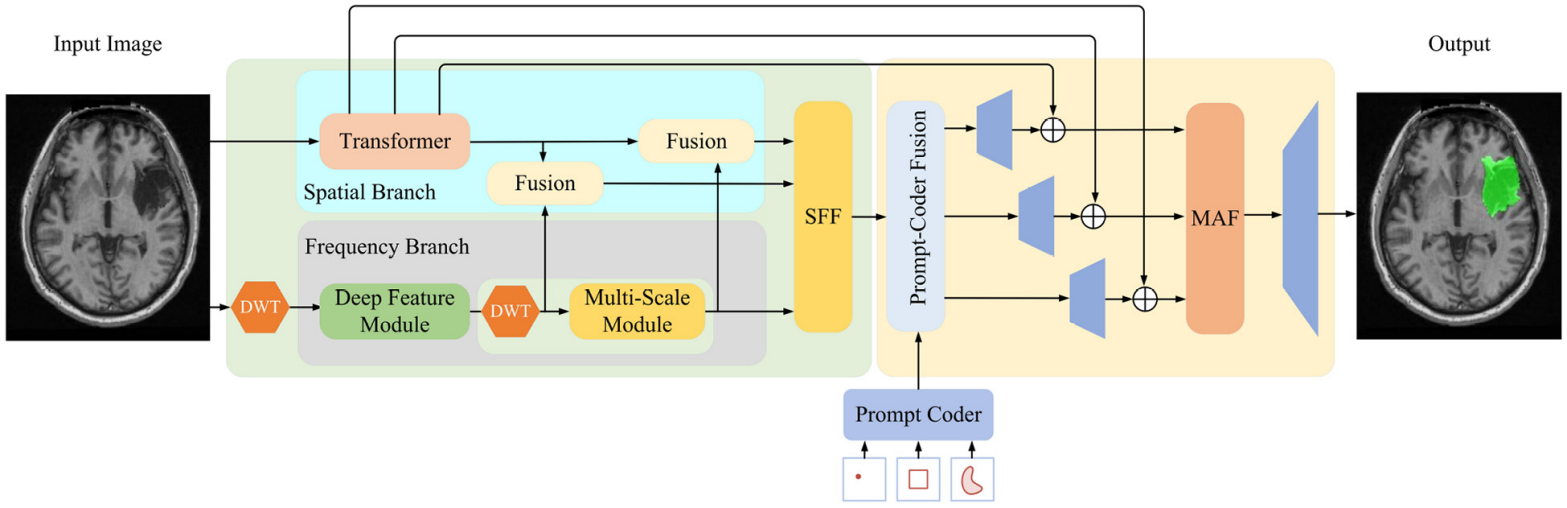
Architecture of the proposed Dual-Branch Encoder Network (DBENet) for brain lesion segmentation.

Figure 2 illustrates the proposed Dual-Branch Encoder Network (DBENet). The model contains a spatial branch and a frequency branch to capture complementary MRI representations. The SFF module integrates spatial-frequency features, whereas the MAF module refines multi-scale lesion representations for segmentation decoding.

Given an input brain MRI image *I*∈ℝ^*C*×*H*×*W*^, DBENet first constructs two complementary feature streams. The spatial branch extracts anatomical context through a Transformer encoder. A frequency-interaction unit is embedded into each self-attention block to enhance boundary-aware responses. The frequency branch captures multi-scale frequency patterns and fine structural details through hierarchical convolutional encoding.

The two streams are integrated by the Spatial and Frequency Fusion (SFF) module. This step generates a unified spatial-frequency representation for lesion structure modeling. After SFF, DBENet adapts SAM (30, 31) by retaining only its prompt encoder. The prompt coder derives foreground, background, and bounding-box prompts from the fused spatial-frequency representation. The retained SAM prompt encoder converts these prompts into prompt embeddings. These embeddings provide lesion-aware localization priors for Multi-scale Attention Fusion (MAF)-based refinement.

The prompt-guided spatial-frequency representation is then refined by the MAF module. MAF strengthens multi-scale contextual modeling and improves boundary refinement before final segmentation prediction. Therefore, the overall information flow consists of dual-branch feature extraction, SFF-based spatial-frequency integration, prompt generation, and MAF-based segmentation decoding. The final segmentation output is formulated in Equation 1 as:


$$\begin{array}{l} F_{h}=F_{\text{SFF}}\left(F_{p},F_{u},F_{l}\right),\;P_{e}=E_{\text{SAM}}^{\text{prompt}}\left(P\right),\;Ŷ=D_{\text{MAF}}\left(F_{h},P_{e}\right), \end{array}$$ (1)

where Ŷ denotes the predicted segmentation map. *F*_*p*_ and *F*_*u*_ represent spatial and frequency features, respectively. *F*_*l*_ denotes the low-frequency structural prior. *F*_*h*_ is the fused spatial-frequency representation generated by SFF. *P* denotes the lesion-aware prompts generated by the prompt coder. *P*_*e*_ denotes the prompt embeddings produced by the SAM prompt encoder. *D*_MAF_ performs prompt-guided multi-scale segmentation decoding.

Specifically, the SFF module is responsible for spatial-frequency feature integration, whereas the MAF module performs multi-scale attention-based refinement. Their detailed designs are presented in the following subsections.

### 3.2 Spatial and Frequency Fusion (SFF) module

**Figure 3:**
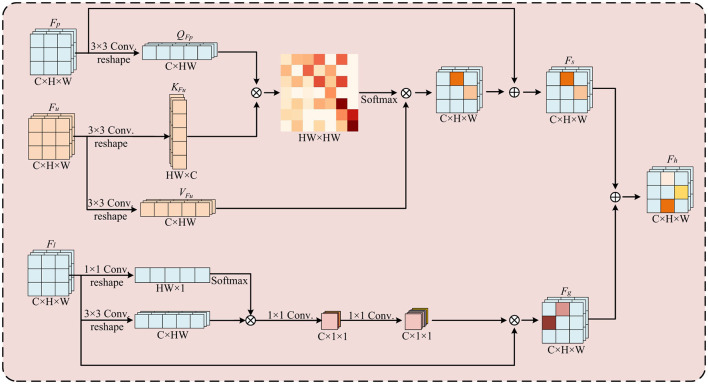
Architecture of the proposed SFF module.

The SFF module integrates spatial and frequency representations to enhance lesion-specific features, as shown in Figure 3. It receives three feature tensors: the spatial feature $F_{p}\in {\mathbb{R}}^{C\times H\times W}$, the frequency feature $F_{u}\in {\mathbb{R}}^{C\times H\times W}$, and the low-frequency fused feature $F_{l}\in {\mathbb{R}}^{C\times H\times W}$. *F*_*p*_ is derived from the spatial branch, whereas *F*_*u*_ is obtained from the frequency branch. *F*_*l*_ provides low-frequency structural priors aggregated across the two streams. Here, *C* denotes the number of channels, while *H* and *W* denote the height and width of the feature map.

To model cross-domain interactions, SFF projects *F*_*p*_ and *F*_*u*_ into query, key, and value representations. Specifically, *F*_*p*_ is projected into the query matrix *Q*_*F*_*p*__. In contrast, *F*_*u*_ is projected into the key matrix *K*_*F*_*u*__ and value matrix *V*_*F*_*u*__. This design allows spatial contextual cues to interact with frequency-aware boundary information in Equations 2 and 3:


$$\begin{array}{l} T\left(F^{*}\right)=\text{Reshape}\left({\text{Conv}}_{3\times 3}\left(F^{*}\right)\right), \end{array}$$ (2)

$$\begin{array}{l} Q_{F_{p}}=T\left(F_{p}\right),\;K_{F_{u}}=T\left(F_{u}\right),\;V_{F_{u}}=T\left(F_{u}\right), \end{array}$$ (3)

where F^*^ denotes the feature tensor to be projected, which can be *F*_*p*_ or *F*_*u*_. T(·) represents a projection operation consisting of a 3 × 3 convolution and a reshape operation. *Q*_*F*_*p*__ is derived from the spatial feature. *K*_*F*_*u*__ and *V*_*F*_*u*__ are derived from the frequency feature. The convolutional projections for *Q*_*F*_*p*__, *K*_*F*_*u*__, and *V*_*F*_*u*__ have independent learnable parameters.

Based on the projected features, SFF first performs spatial-frequency cross-attention. The key matrix *K*_*F*_*u*__ interacts with the query matrix *Q*_*F*_*p*__ to generate an attention map. This map captures spatial correlations guided by frequency-domain boundary responses. The attention map is then multiplied by *V*_*F*_*u*__, yielding a frequency-enhanced representation in Equation 4:


$$\begin{array}{l} A\left(Q_{F_{p}},K_{F_{u}},V_{F_{u}}\right)=V_{F_{u}}⊗\text{SoftMax}\left(K_{F_{u}}\cdot Q_{F_{p}}\right), \end{array}$$ (4)

where A(·) denotes the spatial-frequency cross-attention function.

The enhanced representation is then combined with the spatial feature *F*_*p*_ to generate the fused feature *F*_*s*_ in Equation 5:


$$\begin{array}{l} F_{s}=A\left(Q_{F_{p}},K_{F_{u}},V_{F_{u}}\right)⊕F_{p}, \end{array}$$ (5)

where *F*_*s*_ denotes the fused spatial-frequency feature. ⊕ denotes element-wise addition.

In parallel, the low-frequency feature *F*_*l*_ is processed by a channel-attention branch. This branch emphasizes informative structural responses and produces *F*_*g*_ in Equation 6:


$$\begin{array}{l} F_{g}=\mathrm{CA}\left(F_{l}\right), \end{array}$$ (6)

where *F*_*g*_ denotes the channel-attended low-frequency feature. CA(·) is the channel attention function.

Finally, the SFF output *F*_*h*_ is obtained by combining the cross-attention feature *F*_*s*_ and the channel-attended feature *F*_*g*_ in Equation 7:


$$\begin{array}{l} F_{h}=F_{g}⊕F_{s}, \end{array}$$ (7)

where $F_{h}\in {\mathbb{R}}^{C\times H_{1}\times W_{1}}$ denotes the output of the SFF module. This output is forwarded to the subsequent prompt-guided and multi-scale refinement stages.

### 3.3 Multi-scale Attention Fusion (MAF) module

**Figure 4:**
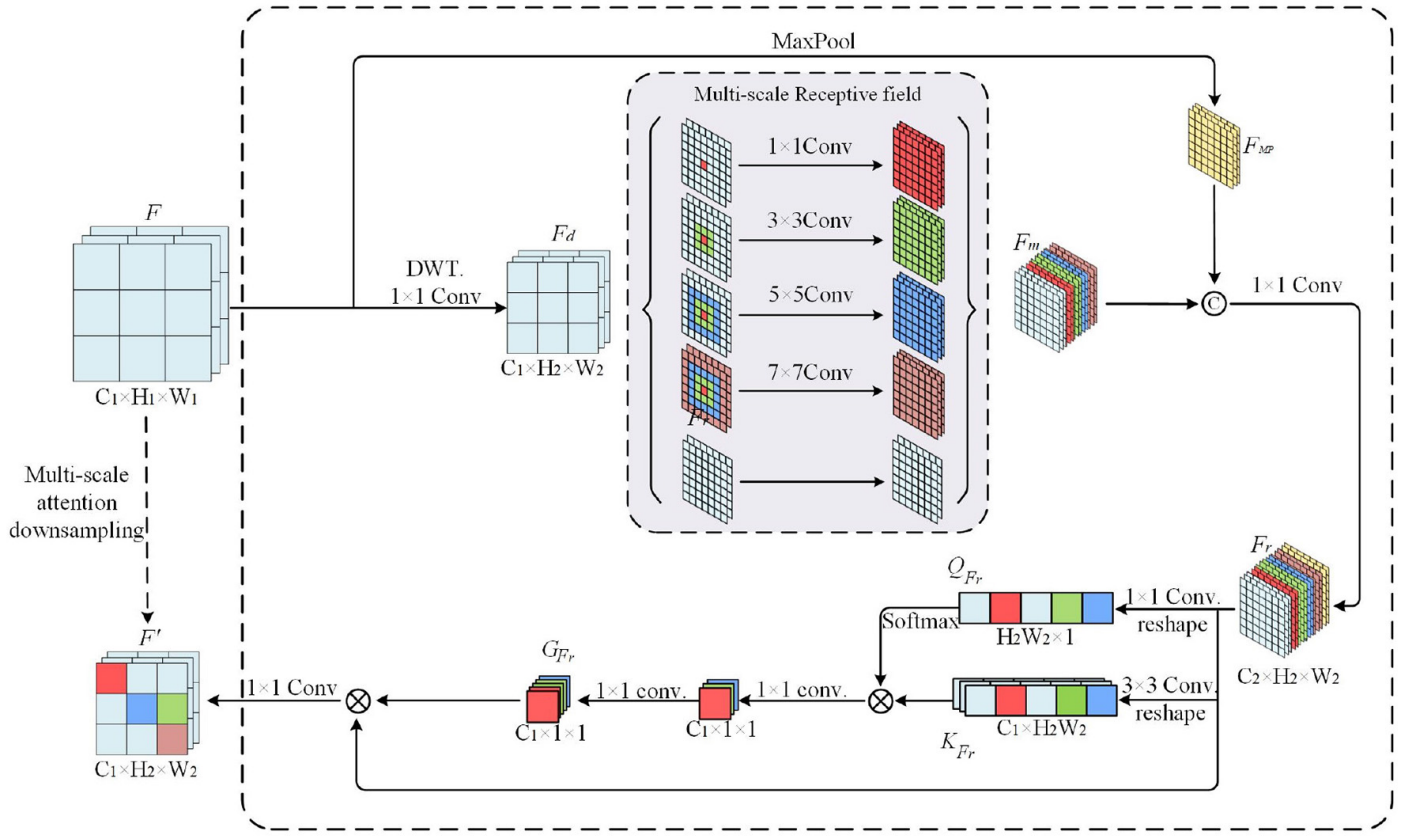
The architecture of the proposed Multi-scale Attention Fusion (MAF).

The MAF module refines lesion features by integrating multi-scale spatial cues and frequency-aware representations, as shown in Figure 4.

Multi-scale decomposition: Given an input feature map *F*, MAF first applies max-pooling and Discrete Wavelet Transform (DWT). These operations generate a down-sampled spatial feature *F*_*MP*_ and a low-frequency representation *F*_*d*_ in Equations 8 and 9, respectively:


$$\begin{array}{l} F_{MP}=\text{MaxPool}\left(F\right), \end{array}$$ (8)

$$\begin{array}{l} F_{d}=\psi \left(F\right), \end{array}$$ (9)

where ψ(·) denotes the DWT operation.

To enrich multi-scale representations, the low-frequency feature *F*_*d*_ is forwarded to a multi-scale receptive-field branch. In this branch, parallel convolutions with kernel sizes 1 × 1, 3 × 3, 5 × 5, and 7 × 7 are applied to *F*_*d*_. The resulting feature maps, together with the original low-frequency feature, are concatenated to form the multi-scale representation *F*_*m*_. This design enables the module to capture lesion patterns at different spatial extents while preserving low-frequency structural information.

The down-sampled spatial feature *F*_*MP*_ is then concatenated with *F*_*m*_ to construct the unified representation in Equation 10:


$$\begin{array}{l} F_{r}=F_{MP}\text{©}F_{m}, \end{array}$$ (10)

where © denotes the concatenation operation. $F_{r}\in {\mathbb{R}}^{C_{2}\times H_{2}\times W_{2}}$ integrates down-sampled spatial context and frequency-aware multi-scale information. Although its spatial resolution is reduced, *F*_*r*_ retains rich semantic cues for subsequent attention refinement.

To adaptively emphasize informative channels, MAF applies channel attention to the unified representation *F*_*r*_. The attention operation generates a channel-wise attention map *G*_*F*_*r*__ in Equation 11:


$$\begin{array}{l} G_{F_{r}}=\mathrm{CA}\left(F_{r}\right), \end{array}$$ (11)

where CA(·) denotes the channel attention function.

The attention map *G*_*F*_*r*__ reweights *F*_*r*_ to enhance lesion-relevant responses. A 1 × 1 convolution is then applied to align the channel dimension and produce the refined MAF output by using Equation 12:


$$\begin{array}{l} F^{\prime }={\text{Conv}}_{1\times 1}\left(F_{r}⊗G_{F_{r}}\right), \end{array}$$ (12)

where ⊗ denotes element-wise multiplication. The output *F*′ encodes refined multi-scale features with enhanced lesion sensitivity.

### 3.4 Loss function

After spatial-frequency fusion and multi-scale refinement, DBENet is trained with joint region-level and pixel-level supervision. We formulate the optimization objective as a hybrid loss that combines Dice loss and Binary Cross-Entropy (BCE) loss as defined in Equation 13:


$$\{\begin{array}{c} L_{\text{Total}}=L_{\text{Dice}}+L_{\text{BCE}}\text{ (Total loss)}\\ L_{\text{Dice}}\;=1-\frac{2|X\cdot Y|}{\left|X|+|Y\right|}\text{ (Dice loss),}\\ L_{\text{BCE}}\;=-\sum [(1-Y)\log(1-X)+Y\log X]\text{ (BCE loss)} \end{array}$$ (13)

where *X* and *Y* denote the predicted segmentation map and the corresponding ground-truth label, respectively. The Dice loss promotes region-level overlap between the prediction and ground truth. The BCE loss provides pixel-level supervision by penalizing pixel-wise classification errors. Their combination improves lesion-region consistency and local prediction accuracy, leading to more stable segmentation optimization. The training procedure of DBENet is summarized in Algorithm 1.

Algorithm 1 Training procedure of DBENet. 
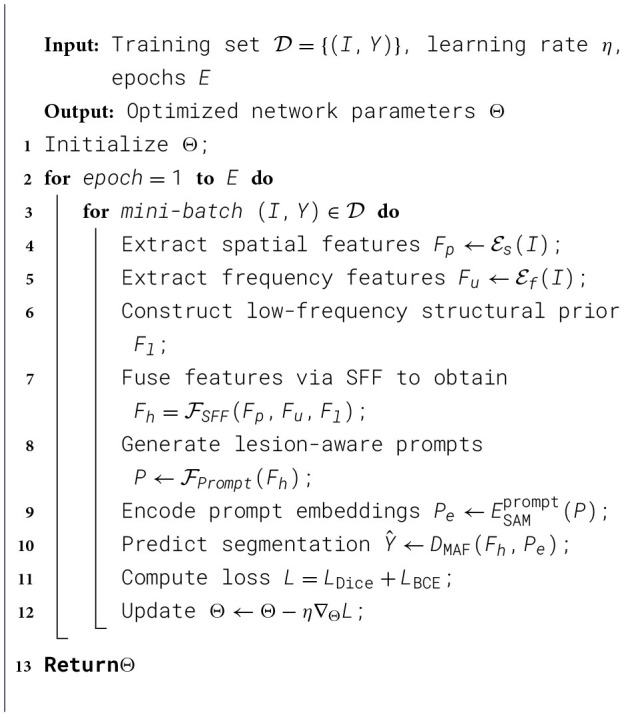

## 4 Experiment

### 4.1 Datasets and implementation details

*(1) Datasets:*

**Figure 5:**
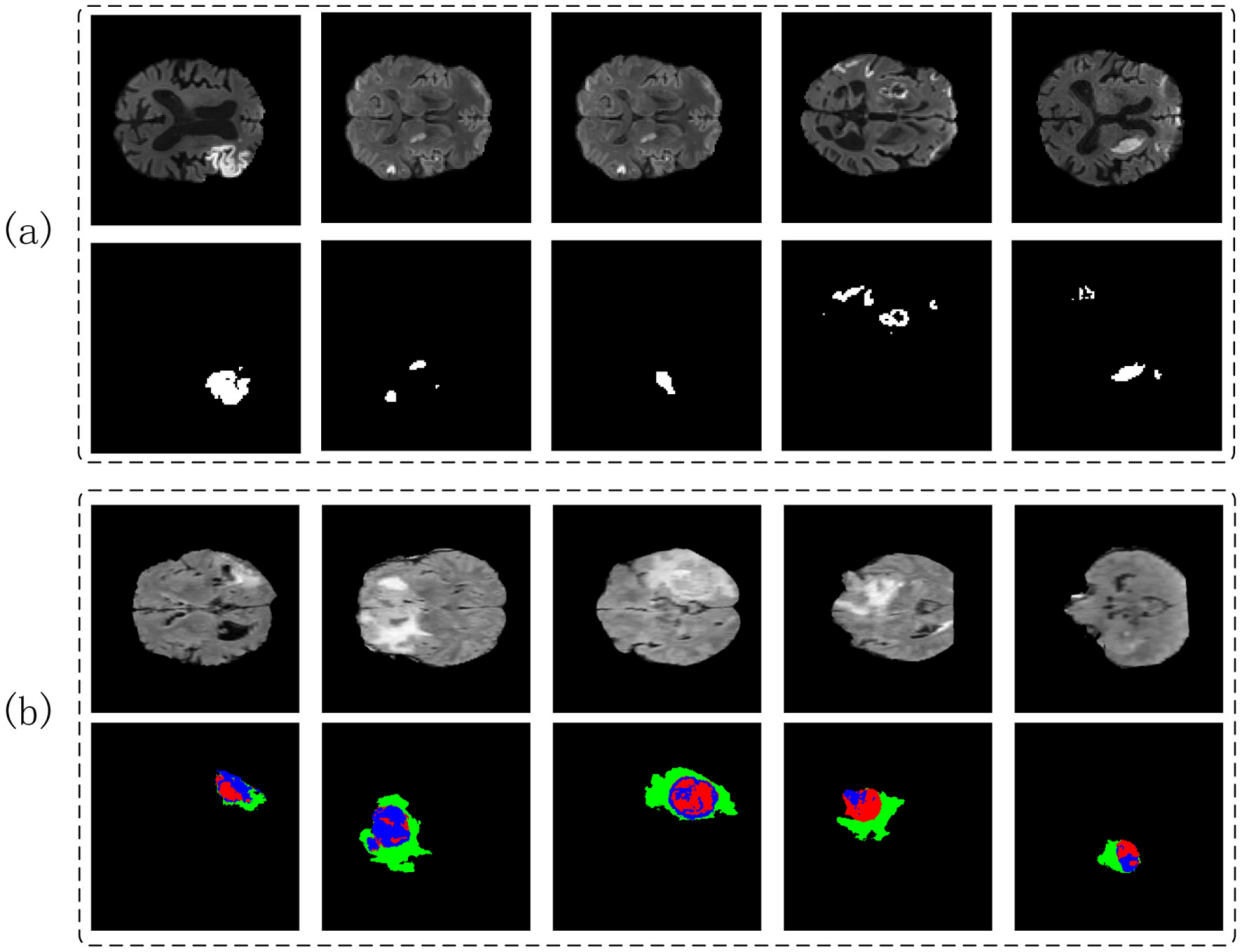
Samples and ground-truth annotations from the two datasets. **(a)** Samples (first row) and corresponding ground truth (second row) from the ISLES 2022 dataset. **(b)** Samples (first row) and corresponding ground truth (second row) from the BraTS 2018 dataset.

• ISLES 2022 Dataset: The ISLES 2022 dataset (32) is a widely used benchmark for acute ischemic stroke lesion segmentation from multimodal MRI (Figure 5a). Each case provides three complementary MRI modalities, namely Diffusion Weighted Imaging (DWI), Apparent Diffusion Coefficient (ADC), and Fluid-Attenuated Inversion Recovery (FLAIR), which capture different pathological characteristics of ischemic lesions. Lesions in this dataset vary substantially in size, shape, location, and image contrast across patients. Differences in acquisition protocols and scanner vendors further increase the difficulty of accurate lesion delineation. These characteristics make ISLES 2022 a valuable benchmark for evaluating robustness and generalization in brain lesion segmentation.

• BraTS 2018 Dataset: The BraTS 2018 dataset (33) is a representative benchmark for brain tumor segmentation based on multi-parametric MRI (mpMRI). To reflect the heterogeneity encountered in clinical practice, the dataset incorporates MRI scans acquired from multiple institutions using different imaging protocols and scanner configurations. As shown in Figure 5b, ground-truth labels are provided for tumor sub-regions. The dataset includes multiple structural MRI modalities that have been preprocessed and standardized to a common anatomical space and resolution. Due to the complex tumor morphology, heterogeneous appearance, and ambiguous boundaries between tumor tissues and surrounding healthy structures, BraTS 2018 remains a challenging benchmark for assessing the effectiveness and robustness of brain lesion segmentation algorithms.

*(2) Implementation Details:* All experiments are conducted on the ISLES 2022 and BraTS 2018 brain lesion segmentation datasets using a PyTorch implementation trained on an NVIDIA RTX A4000 GPU with 16 GB memory. The network is optimized with the Adam optimizer, using an initial learning rate of 5 × 10^−4^ and a weight decay of 10^−3^. To improve generalization, data augmentation including random cropping, horizontal flipping, and rotation is applied. Training is performed on 2D axial slices extracted from the original 3D MRI volumes, each resampled to 256 × 256 pixels and treated as an independent sample, a strategy that reduces computational complexity while preserving anatomical and pathological information. Therefore, DBENet follows a 2D slice-based segmentation framework rather than explicit 3D volumetric segmentation. Inter-slice dependencies are not directly encoded in the current implementation. To partially mitigate this limitation, the architecture strengthens in-slice contextual representation. The Transformer branch models long-range anatomical relationships within each slice. The frequency branch, SFF, and MAF preserve boundary-sensitive and multi-scale structural cues. These designs can improve slice-level consistency by learning recurring anatomical and lesion patterns across training volumes. However, they cannot replace direct volumetric context from adjacent slices. The same training parameters are adopted for both datasets.

To evaluate performance, the proposed DBENet is compared with several representative methods, including U-Net, U-Net++, AttUNet, MEGANet (34), MFMSNet (35), and MedSAM2D (36), all trained and tested under the same experimental settings to ensure a fair comparison. We split each dataset into three subsets for training, validation and testing according to the proportion of 8:1:1.

*(3) Evaluation Metrics:* To objectively evaluate segmentation performance, we report Sensitivity (SEN), Specificity (SPE), Dice coefficient (DICE), and Accuracy (ACC). Statistical significance is assessed using two-tailed t-tests on Dice coefficients, comparing DBENet with each baseline on each dataset. The related definitions are provided in Equations 14–17:


$$\begin{array}{l} Sensitivity\left(SEN\right)=\frac{TP}{TP+FN}, \end{array}$$ (14)

$$\begin{array}{l} Specificity\left(SPE\right)=\frac{TN}{TN+FP}, \end{array}$$ (15)

$$\begin{array}{l} Dice\;Coefficient\left(DICE\right)=\frac{2\times TP}{2\times TP+FP+FN}, \end{array}$$ (16)

$$\begin{array}{l} Accuracy\left(ACC\right)=\frac{TP+TN}{TP+TN+FP+FN}, \end{array}$$ (17)

where *TP* (True Positives) is the number of lesion pixels correctly predicted as brain lesions, *TN* (True Negatives) is the number of non-lesion pixels correctly identified as non-lesions, *FP* (False Positives) refers to non-lesion pixels incorrectly predicted as brain lesions, and *FN* (False Negatives) refers to lesion pixels incorrectly predicted as non-lesions.

### 4.2 Quantitative evaluation

Table 1 presents a comprehensive quantitative comparison of different segmentation models on the ISLES22 and BraTS18 datasets. The compared methods are selected to cover representative segmentation paradigms. U-Net, U-Net++, and AttUNet represent classical CNN-based encoder–decoder architectures. MedSAM2D represents foundation-model-based medical image segmentation. MEGANet and MFMSNet represent recent advanced architectures with attention mechanisms, edge guidance, and multi-scale feature fusion. This selection enables a balanced comparison across conventional, foundation-model-based, and recent task-specific segmentation methods. DBENet is designed to address three challenges that remain insufficiently handled by these methods: weak lesion contrast, ambiguous boundaries, and incomplete exploitation of frequency-domain structural cues.

**Table 1:** Performance comparison of different models on the ISLES22 and BraTS18 datasets.

Model	ISLES22					BraTS18				
	SEN	SPE	DICE	ACC	*p*-value	SEN	SPE	DICE	ACC	*p*-value
U-Net	0.7520	0.9994	0.7434	0.9595	< 0.001	0.7011	0.9536	0.7369	0.9367	< 0.001
U-Net++	0.6838	0.9463	0.6871	0.9569	< 0.001	0.6814	0.9503	0.6993	0.9449	< 0.001
AttUNet	0.7107	0.9998	0.7528	0.9601	< 0.001	0.7013	0.9708	0.7388	0.9501	< 0.001
MedSAM2D	0.7364	0.9981	0.7309	0.9636	< 0.001	0.7299	0.9789	0.7416	0.9517	< 0.001
MEGANet	0.8107	0.9991	0.7869	0.9661	< 0.001	0.7766	0.9813	0.7609	0.9606	< 0.001
MFMSNet	0.7191	0.9997	0.7572	0.9733	< 0.001	0.7081	0.9801	0.7551	0.9693	< 0.001
Ours	**0.8657**	**0.9999**	**0.8621**	**0.9811**		**0.8103**	**0.9891**	**0.8261**	**0.9781**	

Best results are in bold. Each p-value denotes a two-tailed paired *t*-test on case-level Dice coefficients between DBENet and the corresponding baseline.

As shown in Table 1, DBENet achieves the highest values across all reported metrics on both datasets. On ISLES22, DBENet obtains SEN, SPE, Dice, and ACC values of 0.8657, 0.9999, 0.8621, and 0.9811, respectively. On BraTS18, it achieves corresponding values of 0.8103, 0.9891, 0.8261, and 0.9781. Among the competing methods, MEGANet shows the strongest overall performance. Its Dice scores are 0.7869 on ISLES22 and 0.7609 on BraTS18. Compared with MEGANet, DBENet improves the Dice score by 0.0752 and 0.0652 on the two datasets, respectively. The paired statistical tests further show significant Dice improvements over all compared baselines.

The quantitative results also reveal the limitations of existing methods. Classical CNN-based methods, including U-Net, U-Net++, and AttUNet, achieve relatively high specificity but lower sensitivity and Dice scores. U-Net and U-Net++ benefit from encoder–decoder structures and skip connections. These designs preserve low-level spatial details but provide limited long-range contextual modeling. AttUNet introduces attention-based feature refinement, but its improvement over U-Net remains limited. This finding suggests that spatial attention alone is insufficient for lesions with weak contrast, irregular morphology, and ambiguous boundaries.

Recent advanced methods, including MedSAM2D, MEGANet, and MFMSNet, provide stronger representation learning. MedSAM2D benefits from segmentation pre-training and prompt-guided localization. However, its general-purpose representation may not fully capture lesion-specific spatial-frequency characteristics in low-contrast brain MRI. MEGANet benefits from multi-scale feature extraction and attention-guided fusion. Nevertheless, it still faces challenges in delineating lesions with heterogeneous intensity distributions and indistinct boundaries. MFMSNet further improves feature representation through multi-scale feature fusion. However, its limited global–local interaction restricts its ability to exploit complementary contextual representations.

The improvements achieved by DBENet are consistent with its design motivation. First, the dual-branch architecture jointly models spatial and frequency-domain features. This design captures complementary cues for boundary delineation and structural detail representation. Second, SFF integrates heterogeneous spatial and frequency features to strengthen lesion-specific representations. Third, MAF combines wavelet decomposition, multi-scale convolutions, and channel attention to enhance discriminative lesion features. These components directly target weak lesion contrast, ambiguous boundaries, and insufficient frequency-domain modeling. Consequently, DBENet provides more accurate segmentation for lesions with irregular shapes, varying sizes, and low contrast.

### 4.3 Model complexity comparison

Table 2 presents a comparison of computational complexity (GFLOPs) and parameter counts of various brain lesion segmentation models at a resolution of 256 × 256. Classical architectures such as U-Net and U-Net++ are computationally efficient, requiring only 13.71–20.53 GFLOPs and 7.77–9.16 M parameters. However, their limited representational capacity restricts their ability to capture complex lesion features. In contrast, recent high-capacity models such as MedSAM2D and MFMSNet achieve strong segmentation performance at the expense of substantially higher computational and memory requirements.

**Table 2:** Comparison of model parameters and GFLOPs at 256 × 256 resolution.

Methods	GFLOPs	Parameters (M)
U-Net	13.71	7.77
U-Net++	20.53	9.16
AttUNet	23.61	10.33
MedSAM2D	43.78	38.88
MFMSNet	32.99	52.54
MEGANet	19.39	27.27
DBENet (Ours)	22.32	10.58

The complexity comparison demonstrates that DBENet achieves superior segmentation performance while maintaining moderate computational cost. Specifically, DBENet requires 22.32 GFLOPs and 10.58 M parameters, making it considerably lighter than MedSAM2D and MFMSNet while outperforming them in segmentation accuracy. Compared with MEGANet, DBENet introduces only a modest increase in computational complexity while reducing the parameter count by more than 50%. These results indicate that the proposed architecture provides an effective balance between segmentation accuracy and computational efficiency.

### 4.4 Visual analysis

**Figure 6:**
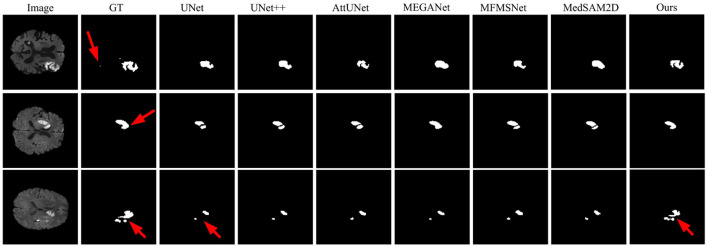
Segmentation results on the ISLES22 dataset. Red arrows indicate representative inaccurate segmentation regions.

**Figure 7:**
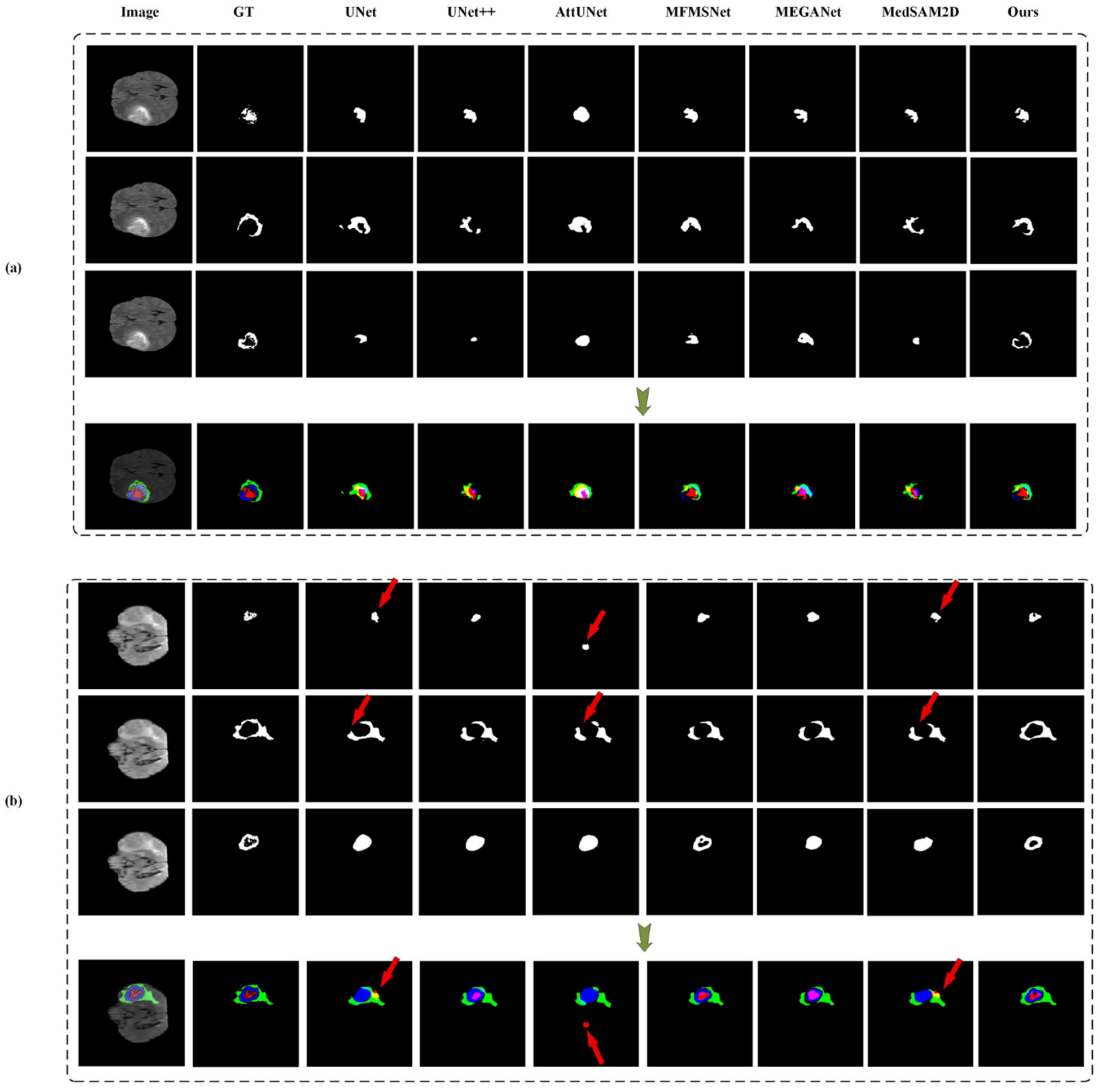
Segmentation results on the BraTS18 dataset. **(a)** and **(b)** show the different segmentation results for samples on different models. There are a few false segmented regions (marked by red arrows). There are few failures except the segmentation of AttUNet in the first row.

To qualitatively evaluate the effectiveness of the proposed DBENet, we compared its segmentation performance with several representative methods, including U-Net, U-Net++, AttUNet, MEGANet, MFMSNet, and MedSAM2D, on two brain lesion segmentation datasets. The visual comparison results are presented in Figures 6, 7. In both figures, the first column shows the input MRI images, while the second column provides the corresponding ground-truth annotations. The remaining columns display the segmentation results generated by DBENet and the competing methods.

As shown in Figures 6, 7, conventional CNN-based architectures, including U-Net, U-Net++, and AttUNet, frequently suffer from over-segmentation and inaccurate boundary delineation. This limitation can be attributed to their relatively simple encoder–decoder structures and direct skip connections, which may introduce redundant or irrelevant features during feature fusion. Consequently, these models often struggle to distinguish lesion regions from surrounding normal tissues, particularly in cases with ambiguous boundaries or heterogeneous lesion appearances.

More advanced approaches, such as MEGANet, MFMSNet, and MedSAM2D, achieve noticeable improvements by incorporating specialized modules for medical image analysis and multi-scale feature modeling. Nevertheless, these methods still encounter difficulties in accurately segmenting complex lesion regions, especially when lesions exhibit irregular shapes, subtle intensity variations, or indistinct boundaries. As a result, under-segmentation, boundary inaccuracies, and loss of fine structural details remain evident in some challenging cases.

The regions indicated by the red arrows highlight typical segmentation failures involving small and irregular lesion structures in Figure 7b. The failure cases demonstrate that, despite the overall strong performance of the model, accurately segmenting extremely small lesions and suppressing spurious predictions in low-contrast regions remain challenging tasks, highlighting the need for further improvements in fine-grained feature representation and contextual modeling. Compared with the competing methods, DBENet generally produces segmentation results that are closer to the ground truth. The proposed method effectively suppresses over-segmentation artifacts while preserving lesion morphology and structural continuity. Moreover, DBENet better captures lesion boundaries and spatial distributions in most challenging cases, leading to more coherent lesion delineation. These results indicate that DBENet is more robust in modeling complex lesion characteristics and provides improved localization accuracy compared with existing methods.

**Figure 8:**
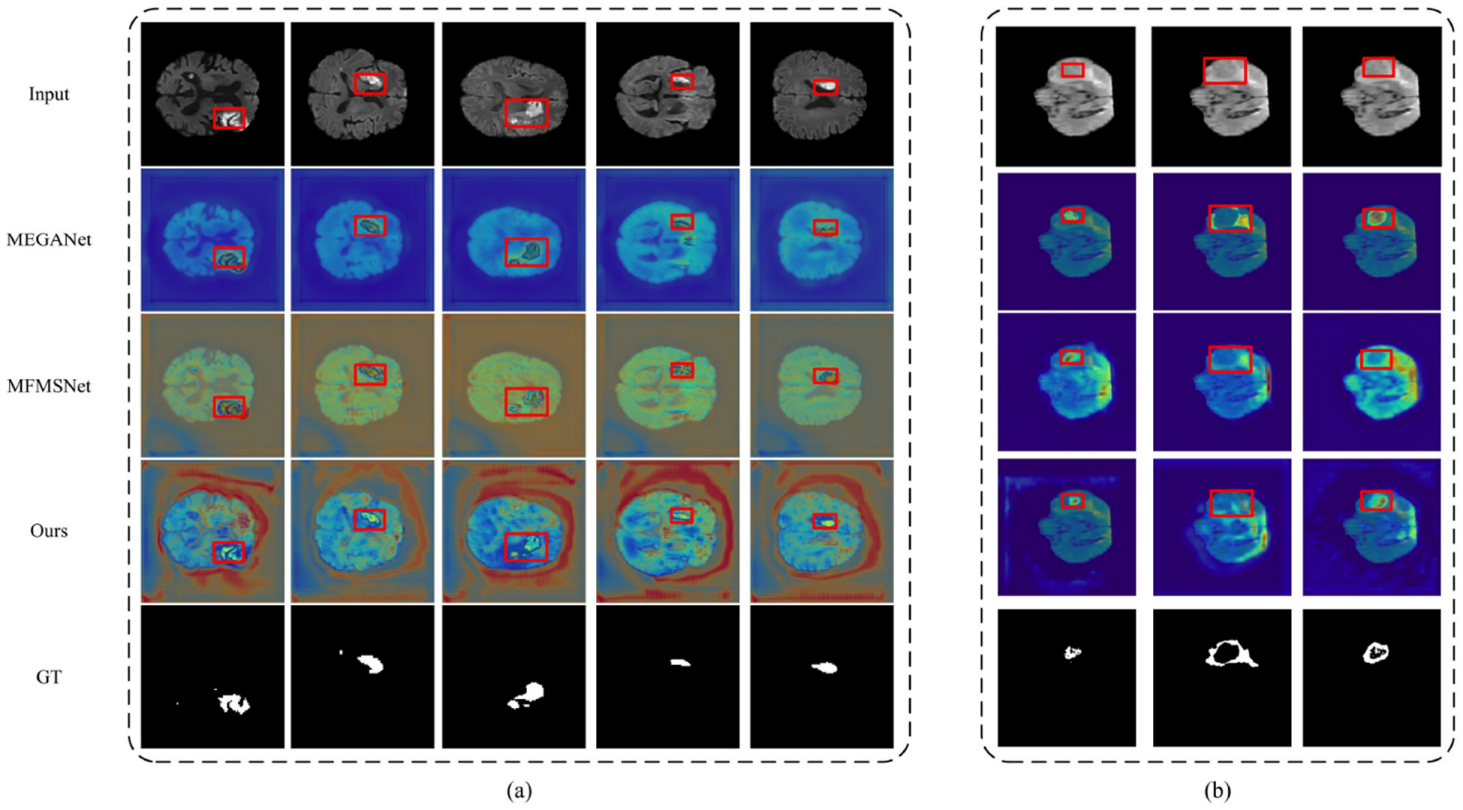
Heat maps on two brain lesion datasets. **(a)** Heat maps on the ISLES22 dataset; **(b)** Heat maps on the BraTS18 dataset. The red boxes in the figure indicate the ground truth (GT) lesion regions.

To further investigate the discriminative capability of different models, Figure 8 presents the corresponding activation heat maps on the two MRI brain lesion datasets. Compared with MEGANet and MFMSNet, DBENet generates more focused and informative responses within lesion regions, while exhibiting clearer boundary awareness and finer structural representation. The heat maps suggest that the proposed model focuses more consistently on lesion regions, resulting in enhanced lesion boundary delineation and better preservation of lesion morphology, particularly in regions with subtle intensity contrasts. These qualitative observations are consistent with the quantitative results reported in the previous section. They further support the improved localization accuracy and robustness of DBENet for brain lesion segmentation.

### 4.5 Ablation study

Table 3 presents the ablation study results designed to evaluate the individual and joint contributions of the Multi-scale Attention Fusion (MAF) and Spatial-Frequency Fusion (SFF) modules on the ISLES22 and BraTS18 datasets. The baseline model corresponds to the network without either module, while subsequent configurations progressively incorporate MAF and SFF. In this ablation setting, MAF and the SAM prompt encoder are activated together because MAF receives prompt embeddings during multi-scale feature fusion.

**Table 3:** Effects of the proposed modules on the ISLES22 and BraTS18 dataset. Best results are in bold.

Dataset	MAF	SAM	SFF	SEN	SPE	DICE	ACC
ISLES22	×	×	×	0.7536	0.9890	0.8020	0.9611
	✓	✓	×	0.8036	0.9810	0.8380	0.9533
	×	×	✓	0.8113	0.9836	0.8299	0.9637
	✓	✓	✓	**0.8657**	**0.9999**	**0.8621**	**0.9811**
BraTS18	×	×	×	0.6993	0.9730	0.7569	0.9631
	✓	✓	×	0.7510	0.9756	0.7937	0.9596
	×	×	✓	0.7639	0.9810	0.8001	0.9633
	✓	✓	✓	**0.8103**	**0.9891**	**0.8261**	**0.9781**

For the ISLES22 dataset, the baseline model achieves a Dice score of 0.8020 and an accuracy of 0.9611. Introducing the MAF module improves the Dice score to 0.8380, demonstrating the effectiveness of multi-scale attention in capturing contextual lesion features across different receptive fields. When only the SFF module is employed, the Dice score increases to 0.8299, indicating that integrating spatial and frequency-domain information facilitates more discriminative feature representation. Notably, combining both MAF and SFF yields the best performance across all evaluation metrics, achieving a Dice score of 0.8621, sensitivity of 0.8657, specificity of 0.9999, and accuracy of 0.9811. Compared with the baseline, the combined model improves the Dice score by 6.0%, highlighting the complementary benefits of the two modules.

A similar trend can be observed on the BraTS18 dataset. The baseline model obtains a Dice score of 0.7569 and an accuracy of 0.9631. Incorporating MAF alone increases the Dice score to 0.7937, while SFF alone achieves a slightly higher Dice score of 0.8001, suggesting that spatial-frequency feature modeling provides stronger standalone discriminative capability. When both modules are integrated, the model achieves the highest performance, with a Dice score of 0.8261, sensitivity of 0.8103, specificity of 0.9891, and accuracy of 0.9781. Relative to the baseline, the combined configuration improves the Dice score by 6.9%, further demonstrating the effectiveness of jointly exploiting multi-scale contextual information and spatial-frequency representations.

Overall, the ablation results consistently validate the effectiveness of the proposed architectural design. Both MAF and SFF contribute positively to segmentation performance when applied individually, while their combination produces the most significant improvements across all metrics and datasets. These findings confirm that MAF and SFF capture complementary information: MAF enhances global contextual understanding through multi-scale attention, whereas SFF strengthens the representation of fine-grained spatial structures and frequency-domain characteristics. Their synergistic integration enables more accurate and robust brain lesion segmentation.

## 5 Conclusion

We propose DBENet, a Dual-Branch Encoder Network for brain lesion segmentation in MR images. DBENet targets heterogeneous lesion appearance, substantial scale variation, and ambiguous boundaries. It also improves the exploitation of complementary feature representations. The network adopts a dual-branch architecture to jointly model spatial- and frequency-domain information. A Spatial and Frequency Fusion (SFF) module effectively combines features from both branches. Meanwhile, a Multi-scale Attention Fusion (MAF) module enhances contextual modeling and refines features across multiple scales.

Extensive experiments conducted on the ISLES 2022 and BraTS 2018 datasets demonstrate the effectiveness of the proposed method. Quantitative results show that DBENet outperforms several representative segmentation approaches, including U-Net, U-Net++, AttUNet, MedSAM2D, MFMSNet, and MEGANet. It achieves Dice scores of 0.8621 and 0.8261 on ISLES22 and BraTS18, respectively. In addition, visual comparisons and activation heat-map analyses confirm that DBENet provides more accurate lesion localization, clearer boundary delineation, and better preservation of lesion morphology. The ablation studies further verify that both the SFF and MAF modules contribute significantly to performance improvement and exhibit strong complementary effects when integrated within the proposed framework. Moreover, DBENet achieves a favorable balance between segmentation accuracy and computational cost, supporting practical implementation under moderate hardware constraints.

Despite these promising results, several limitations remain. DBENet is currently evaluated in a 2D slice-based setting and focuses on binary lesion segmentation. This design reduces memory consumption and computational complexity. However, it does not directly encode inter-slice dependencies in 3D MRI volumes. The learned representations may only indirectly reflect anatomical continuity through recurring slice-level patterns. Future work will extend DBENet toward 2.5D and 3D segmentation by incorporating neighboring slices and volumetric attention. This extension may improve inter-slice continuity and anatomical consistency. We will also extend the framework to richer multimodal clinical information and efficient foundation-model adaptation. These efforts may improve robustness and generalization across diverse imaging protocols and lesion types. Overall, DBENet provides a practical solution for accurate brain lesion segmentation and offers a promising direction for future medical image analysis research.

## Data Availability

Publicly available datasets were analyzed in this study. This data can be found here: http://www.isles-challenge.org/ and https://www.med.upenn.edu/sbia/brats2018/registration.html.
